# 

**Published:** 1895-02-02

**Authors:** 


**Notices of Births, Deaths, and Marriages** are inserted in this column at a charge of 2s. 6d. for an announcement not exceeding four lines.

**Notice to Advertisers and Subscribers.**

All Advertisements, Orders for Copies of The Hospital, and Business Communications should be addressed to **The Manager, NOT TO THE EDITOR,** The Hospital, 428, Strand, London, W.C.

The Cost of Subscription, post free, is as follows:—Per week, 2½d.; per quarter, 2s. 8d.; per half-year, 5s. 3d.; per annum, 10s. 6d. Foreign:—Per Annum, 15s. 2d.; payable, in all cases, in advance.

### NOTICE TO CORRESPONDENTS.

All MS., Letters, Books for Review, and other matters intended for the Editor should be addressed The Editor, The Lodge, Porchester Square, London, W.

The Editor cannot undertake to return rejected MS., even when accompanied by stamped directed envelope.

**"Burdett's Hospital Annual," 1895,**

In Active Preparation.

Sixth year of publication. About 900 pp. Scarlet cloth, gilt lettered. Price 5s. A most complete guide to British, Colonial, Indian, and American Hospitals, Dispensaries, Nursing and Convalescent Institutions, and Asylums. A few copies of the "Annual" for 1894 may still be obtained on application to the Publishers, The Scientific Press (Limited), 428, Strand, London, W.C.

**NOTICE.—The Index for Vol. XVI. (ending September 24th, 1894) is on sale at the Office of this Journal, Price 2½d., post free.**

### SCRAPS AND GLEANINGS.

THE Children's Hospital at Leicester is in excellent financial prosperity, we are glad to learn.

THE Prince of Naples opened a new hospital for sick children at Florence last week.

AN anonymous donor has given the sum of 300 guineas to the Royal Chest Hospital, City Road, E.C.

THE old Foundling Hospital in Rome has been closed, owing to the large mortality amongst the little inmates.

THE Right Hon. Lord Ardilaun has contributed £20 towards the subscription list of the Dublin Hospital for Incurables.

ALDERMAN MEGGITT has generously purchased and provided a site for the proposed Cottage Hospital for Barry and district.

THE authorities of the Newcastle Throat and Ear Hospital are considering the advisability of erecting a new building at a cost of £2,000.

THE question is again under discussion at Montrose Infirmary, whether or not it is advisable to admit infectious cases into the local infirmary!

A LEGACY of £50 has been received from the executors of the Right Hon. Lord Dunsandle at the Dublin National Eye and Ear Hospital, Molesworth Street.

THE *British Medical Journal* recommends that we should daily wash as well as "blow" our noses, at any rate those amongst us who are dwellers in towns.

MRS. CHARLES HALL has given £2,000 to the Chelsea Hospital for Women, in memory of her late husband, Major Charles Hall, formerly of Her Majesty's 1st Life Guards.

DURING the last twelve months there has been 1,747 patients admitted into the Glasgow Convalescent Home, the largest number received in one year and 123 in excess of the admissions in 1893.

THE Metropolitan Benefit Societies' Asylum gave a dinner to the old folk within its walls. The dinner was followed by a presentation of useful gifts, after which a concert and entertainment was held.

WITH the object of assisting the funds of the Free Eye and Ear Hospital at Southampton, the Meister Glee Singers, assisted by other artists, have just given an afternoon concert at the Philharmonic Hall.

BY the will of Mr. Septimus Scott, formerly of the Madras Civil Service, the Tunbridge Wells General Hospital, and the Infirmary, Newtown, Montgomeryshire, benefit by the bequeathal of £100 each respectively.

THE bicycle question is rapidly affecting all sorts and conditions of men and women. We learn that it is now seriously occupying the Pope's attention as to whether it is derogatory to the calling of priests to bicycle.

THE directors of Harrods' Stores (Limited) recommend a dividend of 12 per cent. on the ordinary shares for the six months ending December 31st, 1894, which with the interim dividend paid in July will make 17 per cent. for the year free of income tax.

THE Queen has sent a present of pheasants for the use of the patients in the Cancer Hospital, Brompton. The institution has further been remembered by a lady who gave a tea to the inmates, and also a present to every patient, nurse, and servant.

THE Seamen's Hospital at Greenwich though still possessing an unfortunately large adverse balance on its books, shows this somewhat lessened, we are glad to learn, during the last twelve months of the workings of this excellent institution.

A TOTAL of £492 has been raised through the agency of collecting boxes for the Royal Hospital, Richmond, during the past year. Collecting boxes form a special feature in raising the funds of this hospital, and the result may be regarded as very successful.

THE member of Parliament for Birkenhead has presented a cheque of £106 to the authorities of the Borough Hospital. A contemporary explains this "singular figure," as it terms the gift, by reminding us that 106 was Mr. Lees's majority at the last election.

THE inspection of dwelling houses is not an empty matter of form nowadays. Not only are large numbers of the poorer dwellings in crowded parts of the metropolis consigned to demolition, but in provincial towns, such as Birmingham, the same vigilance is at work.

WHILST a continual appeal to the benevolent is being made on behalf of the Plymouth Royal Eye Infirmary, the Chairman of this institution called attention at the last yearly meeting, to the fact that the precarious nature of a great part of the resources require very careful management to maintain any efficiency within the walls.

IT has been proposed to institute some travelling or caravan infectious diseases hospitals for the north of Northumberland, as well as in Berwickshire. This system has already been adopted in Haddingtonshire, and appears to present less insurperable difficulties (such as site, &c.) than have any other schemes in the proposal to extend the accommodation for fever cases in the district.

INCLUDED in the extensions and improvements which are being affected at the Plymouth Borough Infectious Diseases Hospital at Mount Gould is the provision of a new steam disinfector, which is the first of its class introduced into any town in the west of England. It is claimed that this will prove a profitable investment, while infected articles from dwellings in the town will not be destroyed as heretofore.

THE treasurer of the Royal Hospital for Incurables, Dublin, has received the following intimation from a governor who has always proved himself a generous supporter of the institution: "As the new pavilion at the incurables will necessitate extra expense, I think some effort ought to be made of a special character. I therefore offer to give £100, provided nine other similar subscriptions be contributed before March 31st, 1895."

THE aggregate amount asked for by the special appeal of the Sussex County Hospital appears to be a large one at first sight, £25,000 being required, irrespective of the regular demands of the hospital. But the extension programme is an equally large one. A nurses' home, a hydraulic lift, a new operating room, verandahs on the south side for the accommodation of patients, are among the various improvements which are proposed to be carried out.

### BOOKS RECEIVED.

JOHN BALL AND SONS.
"Probationery Curative Detention." By Norman Kerr, M.D., F.L.S.

HENRY KIMPTON.
"Essentials of Diseases of the Ear." By E. B. Gleason, S.B., M.D.

MESSRS. BAILLIERE, TINDALL, AND COX.

"Bread, Bakehouses, and Bacteria." By Waldo and Walsh.

MESSRS. WILLIAMS AND NORGATE.
"Bad Air and Bad Health." By Harold Wager and Auberon Herbert.

MESSRS. GEORGE PHILIP AND SON.
"How to Live in Tropical India." By J. Murray, M.D.

**Periodicals and Pamphlets.**—English Illustrated, Leisure Hour, Girls' Own Paper, Boys' Own Paper, Light in the Home, Present Day Tracts, Friendly Greetings, Review of Reviews, Holly Leaves, The Princess, Guy's Hospital Gazette, The Therapist, Penny Tales for the People, The Sun, Revue Générale des Sciences, Maandblad voor Ziekenverplajen, The Planter, Christian World Cassell's Family Magazine, Medical Pioneer, Elizabeth, Duchess of Gordon, Provincial Medical Journal, Our Dogs, The Bookseller, Journal de la Santé, Children's League of Pity Paper, The Practitioner, Westminster Review, New York Medical Journal, Public Health, Public Control of Hospitals, Sunday at Home, Reich's Medicinal Anzeiger, London.

### THE STUDENT OF MEDICINE.

### APPOINTMENTS.

C. J. H. Aitken, M.B., C.M., appointed House Surgeon to the Edinburgh Royal Maternity and Simpson Memorial Hospital. A. Ambrose, M.D., LL.D., B.A., B.Ch., D.P.H.Cantab., appointed Medical Officer of Health for the Urban District of Buckhurst Hill. W. H. Bailey, M.B.Lond., M.R.C.S.Eng., appointed Medical Officer for the East Dulwich District of the Parish of St. Giles, Camberwell. V. E. Barrow, L.R.C.P., L.R.C.S.Edin., appointed Medical Officer for the Sampford Peverell District of the Tiverton Union. H. J. Brooksbank, B.A.Cantab., appointed House Surgeon to the Victoria Hospital for Children, Queen's Road, Chelsea. W. H. Bryce, M.B., C.M., appointed House Surgeon to the Edinburgh Royal Maternity and Simpson Memorial Hospital. F. Copeland, M.R.C.S., L.R.C.P., appointed Junior House Physician to the Westminster Hospital. G. Elam, M.D.Dub., B.S., M.R.C.S., L.R.C.P., appointed Honorary Surgeon to the Invalid Asylum, Stoke Newington. J. Elliott, M.D., B.Sc.Lond., appointed Honorary Physician to the Chester General Infirmary. J. Hodges, M.R.C.S.Eng., L.S.A., appointed Senior Medical Officer to the Suffolk General Hospital. E. L. Jacob, M.R.C.S.Eng., appointed Medical Officer of Health for Leatherhead. B. J. James, M.R.C.S.Eng., L.R.C.P.I., appointed Medical Referee to the Hammersmith District of the Royal London Friendly Society, and Medical Officer to the St. Mary's Training College, Brook Green, Hammersmith. P. J. Kingston, M.R.C.S., L.R.C.P.Lond., appointed an Honorary Medical Officer to the Yeovil and District Hospital and Dispensary. J. B. Lawford, F.R.C.S.Eng., appointed Ophthalmic Surgeon to St. Thomas's Hospital. R. Macnab, M.D.Glasg., F.R.C.S.Edin., appointed Honorary Consulting Medical Officer to the Suffolk General Hospital. A. E. Mahood, M.B.R.U.I., M.Ch., appointed Medical Officer for the Northam District of the Bideford Union. Miss Honnor Morten, appointed Lecturer on First Aid and Hygiene, under the Technical Education Board of the London County Council. F. Oliphant, M.B.Edin., appointed Junior House Surgeon to the Chesterfield Hospital. A. C. Pearce, M.B.Lond., appointed House Physician to the Victoria Hospital for Children, Queen's Road, Chelsea. Mr. J. W. Stocker appointed Medical Officer for the No. 6 District of the Romford Union. J. A. M. Thomson, L.R.C.P., L.R.C.S.I., appointed Medical Officer of Health to the Bradford-on-Avon Rural Sanitary Authority. H. S. Walker, F.R.C.S., appointed Lecturer on Ophthalmology and Otology at the Yorkshire College, Leeds. O. M. White, M.R.C.S., L.S.A., appointed Medical Officer to the Royal Naval College, Eltham. J. T. J. Morrison, M.A., M.B., B.C.Cantab., F.R.C.S.Eng., appointed Assistant to the Chair of Surgery in the Mason College, Birmingham.

### VACANCIES.

**Resident Medical Officers.**

Cancer Hospital (Free), Fulham Road, S.W.—House Surgeon. Appointment for six months. Salary at the rate of £50 per annum, with board and residence. Applications to the Secretary by February 4th.

Central London Ophthalmic Hospital, 238A, Gray's Inn Road, W.C.—House Surgeon. Applications to the Secretary by February 12th.

Dewsbury and District General Infirmary, Dewsbury.—House Surgeon, doubly qualified. Salary £80 per annum, with board and residence. Applications to the Chairman of the House Committee by February 6th.

General Hospital for Sick Children, Pendlebury, Manchester.—Junior Resident Medical Officer, doubly qualified. Salary £80 per annum, with board and lodging. Appointment for one year. Applications to the Chairman of the Medical Board by February 6th.

North-Eastern Hospital for Children, Hackney Road, Shoreditch, N.E. —Junior House Physician; doubly qualified. Appointment for six months. No salary, but board and lodging (including washing) provided. Applications to the Secretary, 27, Clement's Lane, E.C., by February 13th.

Staffordshire General Infirmary, Stafford.—House Surgeon, doubly qualified. Salary £100 per annum, with board, lodging, and washing. Appointment for two years, subject to direction of the General Committee. Applications to the Secretary of the Infirmary, Lloyds Bank (Limited), Staffordshire, by February 6th.

**Non-Resident Medical Officers.**

University of Edinburgh.—Additional Examiners in Natural History and Clinical Surgery. Period of office four years. Salary £75 per annum in each case, with £10 per annum for travelling and other expenses in the case of an additional examiner not resident in Edinburgh or the immediate neighbourhood. An additional allowance is made to the Examiner in Natural History for examining for Graduates in Arts. Applications to L. J. Grant, Interim Secretary, University Court, University of Edinburgh, by February 6th.

THE STUDENT OF MEDICINE—*continued.*

Neath Urban District Council.—Medical Officer of Health for the Borough. Salary £30 per annum. Applications to Edwin C. Curtis, Town Clerk, by February 5th.

Suffolk General Hospital, Bury St. Edmunds.—Honorary Assistant Medical Officer. Applications to the Chairman of Committee by February 4th.

